# Editorial introduction

**DOI:** 10.1007/s44265-023-00001-6

**Published:** 2023-03-29

**Authors:** Shujie Yao

**Affiliations:** Li Anmin Institute of Economic Research, Liaoning University, Shenyang, China

**Keywords:** Digital Economy, Sustainable Development, Techonoly, Jiaoning University, China

The launching of this journal, *Digital Economy and Sustainable Development*, signifies the great effort and ambition of Liaoning University to become an academic leader through creating a modern platform for academic exchanges in two important and interconnected fields of economic research in the 21^st^ century: *digital economy* and *sustainable development*.

In this journal, *digital economy* refers to the on-going fourth industrial revolution prevailing in the world driven by all kinds of digital technologies, including artificial intelligence (AI), big data, blockchain, digital automation, and the like. *Sustainable development* refers to social economic development driven by digital technologies with a perspective of improving environmental quality as well as social-economic equality and justice.

This introduction briefly reviews the world industrialization processes from the first industrial revolution originating from Britain in the 1760’s and how they have evolved into the current state that is now dominated by the digital economy. It then defines the concepts of the digital economy and sustainable development as well as their interconnection. It aims to provide some suggestions and ideas of how research may be directed towards the topics of interest that this journal will focus on to achieve its intended goals and ambition. 

## Evolution of the world industrialization processes

Since Britain started the first industrialization revolution featured with steam engines and related technologies from the second half of the eighteenth century, the world economy has experienced more than three hundred years of prodigious and continuous industrialization and social-economic changes, overshadowing the human civilization history dominated by traditional agriculture and courtyard off-farm activities for thousands of years. Technological progress has also brought about pervasive globalization as well as polarization of mankind and geo-economic politics, triggering competition, confrontation and division between different countries, the rich and the poor, the weak and the strong.

Despite her relatively small population and land territory, Britain emerged to become the world’s first economic-military superpower thanks to her position as the global leader in technological progress, which enabled her to engage in world trade, gaining huge economic as well as military strength to colonize more than one-third of the world population.

The second industrial revolution starting from the second half of the nineteen century had much more profound and potent impacts on the world economy and politics as it was participated by a larger number of countries with far more innovations and technological breakthroughs that propelled labor productivity to a much higher level. Electricity, oil, steel, synthetized materials and nuclear power were just some of the most important innovations and products that enabled labor productivity to be improved enormously, allowing people to enjoy a much higher living standard than ever before. The drive to seek superiority in economic and technological competition led the European economic-military powers to sequential large-scale wars, even drawing the US to the battle grounds in the first and second world wars. During these wars, the US garnered from the need of ammunition and weapons by its allies and many important domestic technological innovations. As a result, many colonized countries were able to become independent and the US gradually replaced Britain to be the most powerful economic, technological and military superpower. The US’ dominance in the world economy, international politics, science and technology, and military affairs has lasted more than 100 years up to today.

The third industrialization revolution from after the Second World War, featuring with computing and the internet, have transformed all the industrial production processes. Not only has the productivity of agriculture and manufacturing been greatly improved, the services and financial industries have also become the most dominant sectors of the national economy in the developed world. As communication and transportation costs declined sharply between countries, international trade and cross-border foreign investment have become ever more pervasive between different countries, leading to a far more complicated world order relating to trade, investment and foreign diplomacy. Although large scale wars have been avoided for more than 70 years, smaller scale wars have frequently broken out between the world superpowers and the relatively small and weak countries which share different interests with the former. Technological changes are more than often driven by the desire to maintain hegemony by the rich and powerful nations over the poor and weaker ones. As a result, the first three industrialization revolutions have not led to a more balanced economic development and human livelihood between the developed and less developed economies. Hunger and undernourishment still prevail not only among the least development countries in Africa, Latin America and South Asia, but also among the low-income groups in the high and middle-income countries.

Due to the exploitation and intensive use of fossil fuels as a result of industrialization, the globe has been filled with increasing amount of green-house gases with devastating climate change as well as serious human health implications. This is because of population expansion, relentless production, and non-restrained consumption of agricultural and manufacturing goods, particularly the highly-polluting products such as metals, paper, motor vehicles, cement and chemical products. Although people in the industrialized countries have enjoyed enormously the results of industrialization and productivity growth driven by technological progress, many people in the least developed countries have enjoyed little over the same period of time although they have to passively bear the severe consequences of climate change and all kinds of pollution in the globe. Technological changes and the sequential processes of industrial revolutions led by the rich countries have obvious advantages in raising labor productivity but at the same time they have led to more conflicts between the rich and the poor as well as the weak and strong countries in the world. Consequently, world economic development and technological innovations need to be promoted in such a way that can benefit all people and the natural environment.

## Emergence of the digital economy

The fourth industrial revolution starting from the new century featuring with artificial intelligence and the internet of things has been based on the previous industrialization processes, particularly the third industrialization revolution. It is hoped that with AI, human beings will be able to produce more products and services with relatively less inputs of human effort and materials (Sundarajan 2016; United Nations Conference on Trade and Development 2020; World Economic Forum 2020) For example, we can read books on computer screens or mobile phones, rather than from newspapers. Office workers do not need to travel long distances to work in offices or meet in conference rooms. They can perform their duties and/or join video conferences in their homes. In the last 3 years of the Covid-19 pandemic, it has been proved that working, meeting, learning/teaching at home can effectively replace face-to-face contacts, although the results may still not be as ideal as desired.

Machine learning, blockchain, automatic driving, artificial intelligence (AI) guided diagnosis of patients, robots, drones, and the like, have enabled human beings to do things without much real physical labor inputs. Even space stations and drones are now powered by solar-panels thanks to AI technologies. In theory, the quality of human livelihood and labor productivity could be remarkably increased without much pollution to the natural environment. This is exactly the on-going process of the fourth (new) industrial revolution featured with multiple AI technologies and the internet.

The launch of ChatGPT on November 30, 2022, has sent a powerful shock wave across the globe in recent months. The AI-driven conversation platform attracted more than 100 million users within 2 months, compared for Tik Tok that took 9 months to gain the same magnitude of interest. Many countries are trying their best to produce similar platforms to ChatGPT, just like zoom, Tencent Meeting and the like for video conferencing. China’s Fudan University has launched a trial platform called MOSS which is said to have similar features with ChatGPT although the quality is not yet the same.

Some economic theorists have attributed the pre-industrialized world as the *agrarian society*, the industrialized world as the *industrial society* and the current AI-driven industrialized world as the *digital society*. This is because most new technological innovations are fundamentally based on data, computing and digital techniques. All these changes and their applications in the real world to improve human livelihood and total factor productivity as well as people’s life styles can be simply defined as the *digital economy*.

In other words, digital economy can be referred to economic activities that are enabled by digital technologies, such as the internet, mobile devices, pretrained generative and other digital platforms. In the last two decades, digital economy has fundamentally transformed the way people live, work, and do business, creating numerous opportunities for entrepreneurs and businesses, but at the same time posing new challenges for policymakers as well as the global law and order.

In particular, digital economy has grown rapidly in most recent years, driven by the increasing availability of high-speed internet connections and high-power computing facilities. The latest official statistics report that in China alone, the number of mobile phone users has reached 1.2 billion by the end of January 2023. Nowadays, more and more people in the world are using internet for shopping, communicating, and data mining. This has created opportunities for businesses to reach customers, sell products and services, and expand their operations across national boundaries. Digital economy has also enabled the rapid emergence of new business models, such as the sharing economy and automatic driving. Drones have been widely used for civilian services and military combats. These models allow individuals and firms to monetize their skills, assets, and time in new ways, often through digital platforms. For example, platforms like Didi, Uber, Airbnb, and Tianmao have disrupted traditional industries by connecting people directly with services they need, imposing huge pressure on off-line shopping and employment.

Digital economy has also transformed traditional industries such as manufacturing, healthcare, and finance. For example, people can now go anyway without carrying cash as they can make all kinds of payment using mobile phones. People can also make investments such trading shares and buying other investment products through internet transactions on their phones. Travelers can now buy train (or air) tickets and board on trains (airplanes) using their mobile phones. Air passengers can now even use their mobile phones for normal communications in the air. The use of digital technologies has enabled manufacturers to automate production processes, improve supply chain management, and create new products and services. Healthcare providers are using digital technologies to improve patient care, monitor health outcomes, and reduce costs. Financial institutions are using digital technologies to improve customer experiences, develop new products, and manage risk. Digital economy has also enabled new forms of communication and collaboration, such as video conferencing, social media, and online collaboration tools. These tools have enabled businesses to connect with customers, suppliers, and partners in new ways, irrespective of geographic location.

While digital economy has created different opportunities for individuals and businesses, it has also posed new challenges for policymakers and regulatory authorities. The rise of digital platforms has led to new issues about competition, privacy, and security. For instance, some digital platforms have become dominant players in their respective markets, raising concerns about their impact on competition. Some platforms have also been criticized for their handling of user data, leading to calls for stronger privacy regulations. Abuse of monopolistic powers worsen social income distributions, as people who enjoy such power may become super rich at the expenses of customers and public interests. Digital economy has also created risks and uncertainties related to cybersecurity. As more business and personal data is stored online, the risk of data breaches and cyberattacks may lead to devastating consequences. This requires a much more diligent and effective regulatory environment with international cooperation.

## Sustainable development

For simplicity, sustainable development refers to the process of meeting the needs of the present generation without compromising the ability of future generations to meet their own needs. It is based on the concept of balancing economic growth, social development, and environmental protection to ensure a better quality of life for all people, now and in the future (European Commission 2019; World Commision on Environment and Development 1987; United Nations 2015).

The world is facing a range of environmental, social, and economic challenges that threaten the well-being of people and the planet. These challenges include climate change, biodiversity loss, water scarcity, poverty, inequality, and social unrest. Sustainable development offers a way to address these challenges by creating a more equitable, resilient, and sustainable world.

Sustainable development recognizes that economic growth is necessary to reduce poverty and improve living standards, but it also recognizes that growth must be balanced with social development and environmental protection. This requires a shift away from the traditional model of economic growth that is based on the unsustainable exploitation of natural resources, to a more sustainable model that promotes the efficient use of resources, reduces waste and pollution, and preserves ecosystems and biodiversity.

The United Nations has developed a set of Sustainable Development Goals (SDGs) that provide a framework for global action on sustainable development. The SDGs consist of 17 goals and 169 targets that aim to end poverty, protect the planet, and ensure prosperity for all. The SDGs cover a wide range of issues, including poverty reduction, health, education, gender equality, clean water and sanitation, renewable energy, sustainable cities and communities, responsible consumption and production, and climate action. They recognize that sustainable development requires action at all levels, involving all stakeholders, from individual actions to global cooperation.

Achieving sustainable development requires a transformational change in the way we live, work, and do business. This requires a shift towards more sustainable practices and policies that promote economic growth, social development, and environmental protection. Some useful examples that require national and international actions for sustainable development may include: (1) investment in renewable energy and energy efficiency to reduce greenhouse gas emissions and improve energy security; (2) sustainable agriculture practices that reduce the use of harmful chemicals (e.g., chemical fertilizers and pesticides), conserve soil and water, and promote biodiversity; (3) sustainable urban planning that promotes compact, connected, and walkable cities, with access to public transportation, green spaces, and affordable housing; (4) sustainable business practices that promote responsible consumption and production, ethical supply chains, and corporate social responsibility; (5) pubic policies to ensure fair competition, to benefit the poor, women and children; (6) international cooperation for directed technological innovations that benefit all the countries and all people in the world.

## Interconnection of the digital economy and sustainable development

Digital economy and sustainable development are two critical areas of focus for policymakers, businesses, and society at large. Digital economy offers new opportunities for economic growth and innovation, while sustainable development aims to ensure a more equitable and sustainable future for all people. These two areas are interconnected, and the relationship between them is important for achieving sustainable development goals. This is because digital economy can be a powerful force of sustainable development. For instance, digital technologies can improve access to education, information capturing and healthcare, reduce energy consumption, and enable sustainable transportation, urban planning and rural development. Digital technologies can also facilitate the transition to a more circular economy, where waste is minimized or reused, and resources are used more efficiently. This can be achieved through the use of digital platforms that enable the sharing of goods and services, the use of sensors and data analytics to optimize resource allocation, and the development of more appropriate products and personalized services that are designed for reuse and recycling. Digital technologies are particular useful for central and regional governments for their poverty alleviation campaign and security surveillance in the remote and disadvantaged rural areas where traditional communication infrastructure is poor or non-existent.

Digital economy can also enable new business models that are more sustainable, such as the sharing economy, which enables individuals to monetize underutilized assets, and the circular economy, which aims to reduce waste and promote resource efficiency. These new business models can promote more sustainable consumption patterns and reduce the negative environmental impact of economic activities (Intergovernmental Panel on Climate Change 2018; United Nations Development Programme 2020).

However, digital economy also poses new challenges and risks to sustainable development. For example, the rapid growth of the digital economy can lead to the accumulation of electronic waste, the exploitation of natural resources, and the creation of new forms of inequality. Digital economy also has the potential to exacerbate existing social and economic inequalities, as not everyone has equal access to digital technologies and the benefits they offer. This digital divide can lead to exclusion and marginalization, which can undermine efforts to achieve sustainable development goals.

To ensure that digital economic development contributes to sustainable development, policymakers and businesses must take action to address these challenges and risks. This can be achieved through a range of measures such as: (1) promoting access to digital technologies and digital skills, especially in underserved communities, to reduce the digital divide; (2) encouraging the development of sustainable business models, such as the circular economy and the sharing economy, and promoting sustainable consumption patterns; (3) developing policies and regulations that promote resource efficiency, reduce waste and pollution, and encourage the use of renewable energy sources; (4) fostering innovation and entrepreneurship in digital economic development with a focus on inclusive development goals; (5) setting up appropriate regulatory policies restraining the abuse of monopolistic power at the expenses of the disadvantaged countries, regions, and individuals; (6) training less educated people to better utilize available digital technologies to become more employable and/or to improve their wage-earning ability; (7) paying special attention to rural females, the elderly, and children who may have difficulty in accessing the internet and mobile technology for communication and educational purposes.

Digital economy has emerged as a significant driver of economic growth and innovation in the 21st century. The rapid advancement of digital technologies has transformed the way we live, work, and interact with one another, and this has had a profound impact on the world economy.

One of the most significant impacts of digital economy on the world economy is the increased productivity and efficiency it has brought about. Digital technologies have enabled businesses to streamline their operations and automate many tasks, reducing costs and increasing productivity. This has led to increased output, lower prices for consumers, and higher profits for businesses. Digital economy has also enabled the creation of new business models and industries. The rise of e-commerce, social media, and other digital platforms has created new opportunities for entrepreneurs and businesses to reach customers and expand their markets. Digital economy has also facilitated increased trade and globalization. Digital technologies have enabled businesses to connect with customers and suppliers around the world, breaking down geographical barriers and enabling cross-border trade. This has led to increased competition, lower prices for consumers, and new opportunities for businesses to expand into new markets.

However, digital economy has also had some negative impacts on the world economy. One of the most significant is job displacement, as automation and digital technologies replace traditional jobs in many industries. This can lead to unemployment and underemployment, particularly for workers who lack the skills and education to adapt to new working practices. Furthermore, digital economy has also contributed to social-economic inequality and injustice, as the benefits of digital technologies have not been evenly distributed. Wealth and income have become increasingly concentrated among a small number of individuals and companies, exacerbating existing inequalities and creating new forms of inequality.

To address the potential problems caused by digital economic development, we need a new approach on sustainable development, which should adopt a holistic approach that considers the social, economic, and environmental aspects of development. This approach must take into account the interconnected nature of global challenges, such as climate change, biodiversity loss, and inequality in the new era of technological changes driven by AI and related technological progress and their applications in industries, communities and international affairs. A comprehensive approach to sustainable development must involve all stakeholders, including governments, businesses, civil society, and individuals. It also requires MNCs (multinational corporations) and domestic businesses to assume more environment sustainable development social responsibility.

For example, investments in AI and digital technologies have to incorporate a “green element” in their cost–benefit nexus, ensuring that investments in green and sustainable technologies are essential for achieving sustainable development. This includes investments in renewable energy, sustainable transportation, sustainable agriculture and development of an incentive system for individuals to restrain their consumption habit or behavior. These investments must be made at a large scale and across all sectors of the economy and across national boundaries. Public–private partnerships (PPP) can play a critical role in financing and promoting sustainable investments.

In addition, sustainable consumption and production are essential for achieving sustainable development. Governments, businesses, and individuals alike must adopt sustainable consumption and production practices to reduce waste and greenhouse gas emissions. This includes the adoption of circular economy principles, such as designing products for reuse and recycling, and reducing the consumption of resources. Government pricing policies relating to consumer goods, public utilities such as water and electricity, and motor vehicles are also important.

In the meantime, achieving sustainable development requires social inclusion, social equity and justice, addressing issues relevant to poverty, inequality, and discrimination. Governments must invest in education, healthcare, and social protection programs to ensure that all people have easy and equal access to basic services and opportunities. Businesses must also ensure that their operations and supply chains do not contribute to social injustice and labor exploitation.

International cooperation is also critical for achieving sustainable development. This includes global efforts to address climate change, protect biodiversity, and reduce poverty and inequality. International agreements, such as the Paris Agreement on climate change and the Convention on Biological Diversity, provide a framework for global cooperation. However, more needs to be done to ensure that these agreements are effectively implemented.

In short, sustainable development is a complex and multifaceted challenge that requires a comprehensive and holistic approach. Digital technological development is an effective instrument to facilitate sustainable development. Governments, businesses, civil society, and individuals must work together to achieve the 17 Sustainable Development Goals. However, rich nations and MNCs should be more willing to share their technologies with the least developed countries for common prosperity, to reduce global and regional conflicts and inequality, and to guard against human suffering from abject poverty and undernourishment. National governments should exploit the opportunities offered by digital technologies to boost domestic growth, to be more actively engaged in globalization for the benefits of ordinary residents. This requires investments in green and sustainable technologies, the adoption of sustainable consumption and production practices, the promotion of social inclusion and equity, and international cooperation. By taking these steps, we can create a sustainable and equitable future for all.

## A brief note on this journal

Researchers in all parts of the world are warmly welcome to make contribution to this journal in various aspects. They can submit their latest research outputs relating to the digital economy, sustainable development and any other related issues for publication. They are also welcome to write book comments and brief policy papers as well as to assist us in refereeing and commenting on papers that are submitted to the journal or papers that have been published in the journal. The long term objective of this journal is to become a world leading platform in the above-mentioned fields of research, making useful contribution to a better future of the world economy with better technologies, environmental quality and human livelihood for all people in the world.

## References

[CR1] European Commission. 2019. *The digital economy and society index (DESI) 2019*. https://ec.europa.eu/digital-single-market/en/desi.

[CR2] Intergovernmental Panel on Climate Change. 2018. *Global warming of 1.5°C*. https://www.ipcc.ch/sr15/.

[CR3] Sundararajan A (2016). The sharing economy: The end of employment and the rise of crowd-based capitalism.

[CR4] United Nations Conference on Trade and Development. 2020. *Digital economy report 2020*. https://unctad.org/system/files/official-document/der2020_en.pdf.

[CR5] United Nations Development Programme. 2020. *Sustainable development goals*. https://www.undp.org/content/undp/en/home/sustainable-development-goals.html.

[CR6] United Nations. 2015. *Transforming our world: the 2030 agenda for sustainable development*. https://sustainabledevelopment.un.org/content/documents/21252030%20Agenda%20for%20Sustainable%20Development%20web.pdf.

[CR7] World Commission on Environment and Development. 1987. *Our common future*. https://sustainabledevelopment.un.org/content/documents/5987our-common-future.pdf.

[CR8] World Economic Forum. 2020. *The global competitiveness report 2020*. https://www.weforum.org/reports/the-global-competitiveness-report-2020.

